# Evaluation of the Effectiveness of Selected Extinguishing Agents for Extinguishing Li-Ion Batteries and for Capturing Selected Contaminants

**DOI:** 10.3390/ma19010180

**Published:** 2026-01-03

**Authors:** Anna Rabajczyk, Justyna Gniazdowska, Piotr Stojek, Piotr Mortka, Tomasz Lutoborski

**Affiliations:** Scientific and Research Centre for Fire Protection, National Research Institute, Nadwiślańska 213, 05-420 Józefów, Poland; jgniazdowska@cnbop.pl (J.G.); pstojek@cnbop.pl (P.S.); pmortka@cnbop.pl (P.M.); tlutoborski@cnbop.pl (T.L.)

**Keywords:** Li-ion battery, fire, extinguishing agents, effectiveness, PAH, metals

## Abstract

The production and use of Li-ion batteries (LIBs) is steadily increasing each year, leading to a growing number of battery-powered products. Consequently, the number of chemical hazards associated with the operation and other stages of the life cycle of this type of cell is increasing as well. Therefore, this study examined the impact of selected extinguishing agents for extinguishing Li-ion battery fires—namely, a dedicated extinguishing granulate, a natural sorbent (exfoliated vermiculite), and quartz sand—on the level of heat and released substances. The study determined the emission of heavy metals and polycyclic aromatic hydrocarbons (PAH) into the air during a cell fire, the concentration of the inhalable aerosol fraction, and the concentration of hazardous substances in the extinguishing agent residue. The analysis concluded that quartz sand provides the most effective heat removal and insulation of the battery from the external environment, which also reduces the amount of pollutants released into the environment.

## 1. Introduction

**Figure 1:**
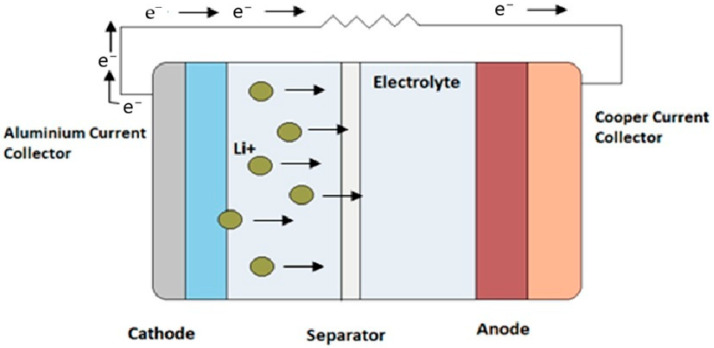
Schematic diagram of the lithium-ion cell (source: own study based on [2]).

The anode, cathode, and electrolyte are main three elements of a battery cell’s structure. Their operating principle involves the movement of Li^+^ in the electrolyte between the cathode and anode, which are placed in a suitable electrolyte (Figure 1). The bidirectional movement of lithium ions between the electrodes is responsible for the cell charging and discharging process [1].

The basic layout of the battery is visible in Figure 1 and includes a metal cathode containing lithium and other metals, such as nickel, manganese, cobalt, iron, or aluminum, depending on its architecture. The most common substances present in the cell are lithium cobalt oxide (LCO), lithium iron phosphate (LFP), lithium manganese oxide (LMO), lithium nickel cobalt aluminum oxide (NCA), lithium nickel manganese (NMC), and lithium titanate (LTO). The most common electrolyte is lithium hexafluoride dissolved in short-chain aliphatic esters of carbonic acid, such as diethyl carbonate (DEC). Anodes can be primarily made of graphite, but other materials such as silicon or lithium titanate can also be used.

Compared to traditional batteries, lithium-ion batteries are characterized by, among other things, higher energy and power density, ease of integration, higher voltages, negligible loss of cell capacity during cyclic charging and discharging, and a wider operating temperature range [3,4]. The high energy density of Li-ion batteries (LIBs) facilitates their widespread use in many industries and sectors. They are used in portable electronics, electric vehicles, and energy storage, as well as the aerospace industry. However, it should be noted that extreme environmental conditions or usage patterns, such as crushing, overcharging, short circuiting, or immersion in seawater, can cause a large amount of chemical energy stored within the battery and contained within a confined space to be suddenly released. This can result in sudden discharge and thermal instability of the system, resulting in fire or even an explosion. To prevent these undesirable effects, researchers are constantly working on solutions to improve the safety of these batteries and accumulators. Some proposed solutions include an internal separator that cuts off the transport of lithium ions between the anode and cathode to interrupt chemical reactions, dendrite-free lithium anodes, and thermally stable cathodes [5,6,7]. The literature also offers suggestions for external solutions, such as thermal management systems [8,9], pulsed current technology, and phase-change materials [10]. An interesting proposal is an aerogel felt material, which, in thermal runaway tests (TR), demonstrates better thermal insulation than, for example, aerogel powder, while minimally reducing the peak temperature [11,12].

The growing use and prevalence of vehicles powered by lithium-ion batteries is leading to a noticeable increase in fire incidence. Currently, electric vehicle sales are estimated to increase to 46.3 million units by 2035 [13], with nearly 1 million electric cars in Poland alone by 2030 [14]. Safety engineering encompasses measures to minimize the likelihood of fire and threat to property, living organisms, and the environment in the event of a fire. In the latter case, access to appropriate extinguishing agents is essential for extinguishing lithium-ion battery fires. Such substances should possess basic properties such as high heat capacity, high wettability, low viscosity, low electrical conductivity, and a positive environmental impact [15]. The most significant problem in LIB fires are the high heat release rate, the complexity of the composition, and the speed of the reaction, which is why the extinguishing agent used to extinguish them should be able to prevent the spread of heat between cells in the module and between modules (cooling effect), as well as inhibit the chemical reactions taking place in the cell (inhibitor) [15,16].

The most commonly used extinguishing agent is water [17]. This substance is characterized by its high heat capacity and latent heat of vaporization and is most often used in the form of water mist, with droplet sizes less than 1000 μm. This droplet size allows for a high surface-to-volume ratio, which allows for the high absorption of heat emitted from the burning material. The literature also reports attempts to protect energy storage facilities using fixed gas extinguishing systems or in combination with water mist [18]. These types of fixed extinguishing systems are commonly used in industry to protect valuable electronics. This approach might be sufficient when applied during an early stage of an incident, especially when it is used along with water mist to obtain a synergistic effect. However, when the fire is already developed, the use of halogenated extinguishing agents to extinguish surfaces maintaining temperatures above 500 °C during fire conditions may provide additional risk and is inconsistent with the manufacturer’s recommendations, as it can cause the thermal decomposition of the extinguishing agent, generating additional hydrogen fluoride. In extreme cases, if adequate ventilation is not provided, this can result in an increased risk to those participating in rescue and firefighting operations and the explosion of accumulated gases [19].

Wastewater may also pose a risk, as water used in the firefighting process can react with the Li compound (i.e., lithium hexafluorophosphate, LiPF_6_), used as an electrolyte, and can produce toxic substances such as HF or flammable substances such as hydrogen. Dissolved forms of various substances, such as metals, polycyclic aromatic hydrocarbons (PAHs), particulate matter (PM), and nanoparticles (NPs), may be present in firewater, posing a significant environmental threat [15,20]. The number of emitted pollutants highly depends on the type of cathode metals used in the construction of the batteries, as well as the boiling point of the carbonic acid esters used as the electrolyte. The emission profile is also influenced by the state of charge (SOC), where a higher SOC typically correlates with higher emissions [21]. In the present work, we tried to check the extinguishing efficiency in the worst-case scenario, and therefore chose to extinguish fully charged NMC cell modules. Further research will likely be necessary to ensure that the obtained results will be similar for batteries with different raw materials, architectures, or SOCs.

It is important to note that the effectiveness and efficiency of the currently employed methods are, in some cases, inadequate to enable rapid fire extinguishing and significantly reduce pollutant emissions. The application of solutions such as water mist, a combination of extinguishing agents with water mist, or a combination of dedicated gases with water mist requires the use of chemicals and water and generates wastewater, which often leads to environmental contamination and requires specialized treatment. The issue of dwindling available water resources of adequate quality for firefighting operations must also be considered. Current efforts primarily focus on minimizing water consumption during firefighting operations and developing the optimal use of existing extinguishing agents. There is still a need to explore new technical and tactical solutions to optimize procedures, which will translate into even greater firefighting efficiency [1,22].

Therefore, this study focuses on the use of commonly available and inexpensive extinguishing agents, such as quartz sand, natural sorbent (exfoliated vermiculite), and extinguishing granulate. These agents were subjected to fire tests to verify their fire extinguishing effectiveness on cylindrical lithium-ion batteries, their ability to absorb heat and reduce fire extinguishing time, and their ability to minimize pollutant emissions into the environment. The parameters of extinguishment effectiveness, air emissions, and pollutant accumulation in residues were assessed together under identical test conditions.

## 2. Materials and Methods

### 2.1. Reagents and Materials

**Figure 2:**
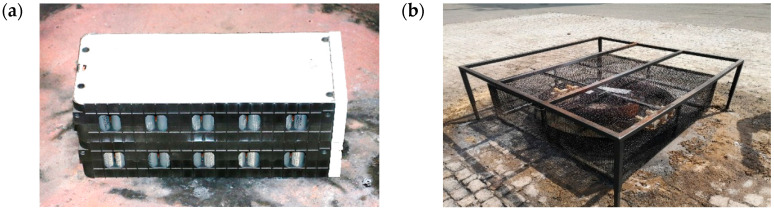
Battery (**a**) and battery fire test stand (**b**) used in the.

Cylindrical lithium-ion batteries were used for the tests—a 16P cell module, nominal voltage of 51.38 V, module capacity of 45.6 Ah (Figure 2a).

The test station (Figure 2b) consisted of a circular fire tray for fire extinguishing efficiency testing according to the EN 3-7 standard [23], covered by a double-layered net installed on the metal frame to protect the area from debris in the event of an explosion.

**Figure 3:**
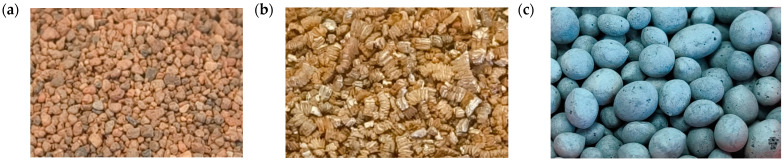
Materials used as extinguishing agents: (**a**) quartz sand (dried), (**b**) natural sorbent—exfoliated vermiculite, (**c**) extinguishing granulate (5×).

The following materials were used for fire extinguishing tests:

- Quartz sand (dried) (Figure 3a)—crystalline powder; grain size 0.1–0.3 mm; composition: SiO_2_ > 97.0%, traces of CaO, MgO, and Fe_2_O_3_; residual moisture content < 0.2% by weight; relative bulk density: approx. 1500 kg/m^3^; grain size: >0.250 mm 0.2%, <0.125 mm 16.4%;
- Natural sorbent—exfoliated vermiculite (Figure 3b)—natural material; molecular formula: Mg_3_(Si_4_O_10_)(OH)_24_H_2_O; maximum moisture content 1.5% (±1%); grain size: <0.125 mm (0.25%), >4 mm (0%); Average bulk density: 116.89 kg/m^3^; Average water absorption: 303%; Average hydrocarbon absorption: 151.1%; Application temperature: −260 °C to +1200 °C;
- Fire extinguishing, transport, and storage granulate (Figure 3c)—inorganic, thermally blown glass granulate based on recycled glass (container glass and flat glass—soda-lime-silicate glass); composition: SiO_2_ 72%, Na_2_O 13%, CaO 8%, Al_2_O_3_ 2%, MgO 3%, K_2_O 1%; solid granules, grain size 1.0–4.0 mm; bulk density 340–460 kg/m^3^.

Chromatographic analyses were performed using analytical standards for the substances under study, including the Certified Reference Material PAH 2000 μg/cm^3^, designated Z-014G, and the Certified Reference Material 1,2,3,4-tetrachloronaphthalene, designated N-005N, supplied by AccuStandard, New Haven, CT, USA. Other reagents, i.e., dichloromethane and hexane, were purchased in analytically pure or higher quality and provided by Merck, San Diego, CA, USA. XAD-2 resin tubes manufactured by SKC, Seoul, Republic of Korea, 6 × 110 mm, 2 sections, 120/60 mg (for PAH sampling). MCE membrane filters, diameter d = 25 mm, pore size 0.22 μm, manufactured by Chemland, Stargard, Poland.

### 2.2. Equipment

To conduct the individual laboratory analyses, control and measurement equipment was used, including the following:

– The battery extinguishing test stand was developed by CNBOP-PIB, Józefów, Poland; it was constructed from a steel frame measuring 2 × 2 m and adjustable in height to approximately 0.7 m, with a safety net between the tray and the frame measuring 1.7 × 1.7 m (Figure 2b);
– K-type sheathed thermocouples rated for temperatures up to 1200 °C;
Data logger/DI-2008 Thermocouple and Voltage Data Acquisition System measurement card, WinDaq Recording Software; DATAQ Instruments, Inc., Aurora, OH, USA;
– Laserliner ThermoSpot-Vision pyrometer with a reading range of −15 °C to +500 °C;
– “Gil Air 3” individual aspirators supplied by Sensidyne LP, St. Petersburg, FL, USA; calibration of the individual aspirators was performed using a “Gilibrator 2” film gas flowmeter supplied by Sensidyne LP, USA;
– Tray dimensions: diameter 53 cm, height 278 cm, volume 71 L, made of stainless steel;
– Shimadzu QC 2010SE gas chromatograph equipped with an AOC 20i+s autosampler and a Zebron ZB-PAH-EU 30 m I.D. 0.25 mm column with a 20 μm film thickness;
– AS 120.R2 Plus analytical balance supplied by Radwag S.A. Radom, Poland;
– 1200 W fireclay heating element for a tiled stove (ceramic heater).

### 2.3. Fire Tests

Two trays were placed on the test stand (Figure 2b): a larger one with a diameter of approximately 1.5 m and a smaller one with a diameter of approximately 0.5 m. The smaller, dry tray was placed in the center of the larger tray. A ceramic heater was used to heat the battery until it self-ignited. The heater was placed in a tray with dimensions: diameter 53 cm, height 278 cm, and volume 71 liters. A Li-ion battery was placed on the heater, and a thermocouple was placed between the heater and the battery. Once the fire had developed, the heater was turned off, and the battery burned spontaneously for 5–6 min, as appropriate for the given test. After 5–6 min of free burning and the intense explosions had subsided, the extinguishing process began. For this purpose, 100 liters of each of the tested agents were prepared, and the tray containing the battery was filled to the top of the tray. Then, for 15 min, observations were made to see if combustion recurred. After the extinguishing test was completed, the top edge of the battery placed in the tray was uncovered by removing the extinguishing material from the battery surface, to check the temperature of the resulting fire debris.

**Figure 4:**
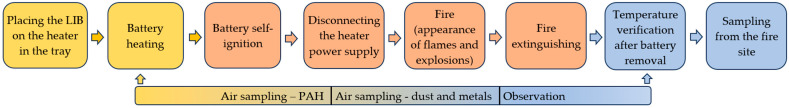
Schematic diagram of the procedure for conducting fire extinguishing tests.

All extinguishing tests were conducted according to the scheme shown in Figure 4 in an enclosed space with dimensions of approximately 13 m × 13 m × 15 m. The experiment was conducted without ventilation to ensure that the concentration of primary pollutants was higher than the limit of quantification (LOQ) of the analytical method. Individual tests were performed for each system.

### 2.4. Physicochemical Testing of Dust and Air Samples

Physicochemical testing included the analysis of dust and air samples collected from the fire zone. For dust analysis, air samples were collected for 60 min in the fire zone using individual aspirators with a flow rate of 2.0 L/min onto cellulose ester membrane filters.

After the specified air sampling time, the membrane filters were subjected to gravimetric analysis and then sealed in plastic containers, protected from damage during transport. Due to the nature of the sample, which contained heavy metal oxides and/or salts, no other forms of sample preservation were used. The sealed samples were forwarded to an external company, the Center for Basic Research, Design and Implementation of Environmental Protection and Biotechnology “OIKOS” Ltd., Environmental Research Laboratory (Święta Katarzyna, Poland), for heavy metal determination.

For PAH analysis, air samples were collected for 60 min using individual aspirators with a flow rate of 1.0 L/min onto XAD-2 synthetic resin tubes. The substances adsorbed onto the resin were then desorbed with hexane, and the resulting solutions were concentrated in a stream of inert gas (nitrogen) and then subjected to chromatographic analysis using the following parameters:

– Injection temperature: 320 °C Pulsed Splitless (1 min, 130 kPa)
– Splitless time: 40 s
– Injection volume: 2 μL
– Carrier gas: helium 2.0 mL/min, constant flow, linear velocity 50.7 cm/s
– Temperature: Isothermal program at 40 °C for 1 min, then increased at a rate of 15 °C/min to 320 °C, maintained for 10 min
– Ion Source Temp.: 260 °C
– Interface temp: 300 °C
– Detector voltage: 1.3 kV
– Solvent cut time: 8 min
– Detector mode: SIM
– Event time: 1 s

The uncertainty of dust and metal analysis estimated according to EN 13890:2010 [24] for the inhalable dust fraction was 17.4%, and the uncertainty of PAH vapor sampling was 3.5%. The Pearson correlation coefficients (R) for the standard curves and the relative standard deviation (RSD) for the individual analytes during the determination of PAHs are summarized in Table 1.

## 3. Results and Discussion

### 3.1. Fire Extinguishing Effectiveness of the Analyzed Agents

**Figure 5:**
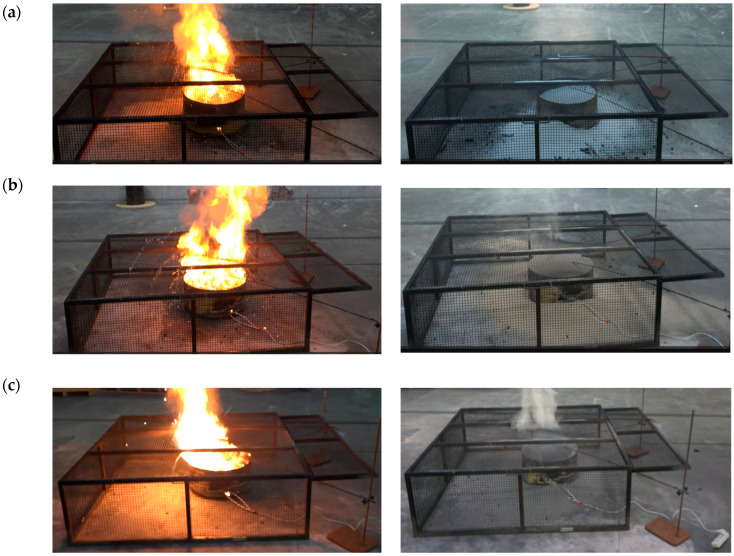
Battery fire and its extinguishing with (**a**) quartz sand, (**b**) exfoliated vermiculite, and (**c**) extinguishing granules.

The ability of an extinguishing agent to stop the uncontrolled heat spread in lithium batteries can be determined based on the cooling efficiency of the agent used [22,25]. Therefore, fire extinguishing tests were conducted on cylindrical lithium-ion batteries, NMC type, which have a 16P module and are characterized by a nominal voltage of 51.38 V and a module capacity of 45.6 Ah. These modules were selected based on data from the literature to determine the scenario that would achieve the most severe environmental impacts related to the release of toxic substances. Using modules with different architectures (especially different cathode materials) or SOC levels could lead to different results. In this preliminary study, only one test was conducted, in accordance with the test conditions. Three inexpensive, readily available materials were used to extinguish the cylindrical lithium-ion battery fires during the fire extinguishing tests: quartz sand, a biodegradable natural sorbent—exfoliated vermiculite—and fire extinguishing granulate (Figure 5). The results of the fire extinguishing tests of lithium-ion batteries using various extinguishing agents are presented in Table 2.

Numerous studies have identified the high heat release rate (HRR) as one of the main problems associated with LIB fires [15]. Furthermore, heat is required to initiate and sustain pyrolysis or fuel vaporization. Therefore, to extinguish a battery fire, the extinguishing agent used must be capable of rapidly absorbing the heat generated during the fire and removing it from the system, preventing heat spread between battery components. The results indicated (Table 1, Figure 5) that each agent used extinguished the fire after approximately 6 min of free burning and prevented further burning for the following 15 min. Quartz sand was found to perform best at heat removal, as evidenced by the lowest post-fire temperature of less than 100 °C and the absence of embers after the battery was exposed. However, when extinguishing granules were used, the highest post-fire temperature of nearly 500 °C and numerous ember clusters were recorded, indicating poor heat capture capabilities. Comparing smoke emissions from the system (Figure 5), it was found that the highest smoke density was observed in the system using the granular extinguishing agent, while the lowest was observed in the system using quartz sand. High smoke emissions, observed during the extinguishing of the battery fire with the fire extinguishing granules, can significantly impact air quality, even in larger areas. The migration of pollutants through the air can lead to environmental contamination not only at the fire site but also pose a health risk to people in the area.

The temperature of the fire site is crucial to the firefighting process due to the possibility of secondary ignition after the initial extinguishment. Numerous embers and high temperatures can ignite plastic parts of cars or other battery-based products or cause the explosion of combustible gases. Therefore, considering firefighting effectiveness and the safety of those involved, quartz sand is the optimal agent among those studied. Sand extinguishing may produce fewer contaminants than water extinguishing, which requires large amounts of water and a long extinguishing time [26]. The effectiveness of agents such as water mist, Firelce, PyroCool, and Stat-X indicates that water-based agents have the best heat capture and cooling properties [26]. Research conducted by Hazard Control Technologies Europe GmbH [27] and Luo et al. [28] indicates that the F-500 agent, formulated as a water-based agent and composed of a hydrophilic and hydrophobic component, enables rapid cooling and prevents reignition. Test results conducted by the US Federal Aviation Administration [25] also indicate that the best results were obtained using water, followed by, in descending order: AF-31, AF-21, A-B-D aqueous solution, and Novec 1230. It was also described in the literature that jet-feeding the agent was significantly less effective in the cooling process [25].

Other works have also used gas-based agents, such as heptafluoropropane and perfluorohexanone (C_6_F_12_O) [29,30], which enable fire control through chemical inhibition, or a combination of perfluorohexanone and fine water mist, which allowed them to achieve a greater fire extinguishing efficiency compared to water mist alone by more than 40% due to the synergy effect [30]. The synergy effect, which allowed for improved extinguishing efficiency, was also achieved by using fine water mist and extinguishing agents in the form of microcapsules, prepared from melamine-urea-formaldehyde resin as the coating and 1,1,1,2,2,3,3,4,4-nonafluoro-4-methoxybutane (C_5_H_3_F_9_O) and 1,1,2,2,3,3,4-heptafluorocyclopentane (C_5_H_3_F_7_) as the composite core. It was found that the microcapsule shell ruptured at 100 °C, allowing the composite extinguishing core to be released within the jet fire, while the fine water mist blocked the transfer of thermal radiation, inhibiting the fire spread. The time required for the battery temperature to drop from peak to low temperature was reduced by 66 s, and the peak temperature of the high-temperature substances above the battery was reduced by 228.2 °C compared to the situation where only water mist was used [31]. When perfluorohexanone and fine water mist were applied under normal pressure conditions, the extinguishing time increased with the increase in the heat release rate from the fire source, and the flame temperature decreased by approximately 300 °C [29]. Compared to these systems, quartz sand and exfoliated vermiculite allow for lower temperatures, not exceeding 200 °C. In the case of quartz sand, this temperature is even 100 °C.

However, it should be noted that these agents are water-based and require the use of chemicals, such as surfactants, which increases the cost of producing the extinguishing agent. The extinguishing agents proposed in this study, with good cooling efficiency, are inexpensive and widely available, as well as environmentally safe, and in the event of post-fire contamination, they can be removed from the fire scene and recycled. Therefore, they constitute an alternative to water or other water-based extinguishing agents.

### 3.2. Pollutant Emissions During a Battery Fire

The chemical composition of pollutants emitted during a fire is determined by the chemical reactions taking place in the burning battery, the most important of which, from the point of view of fire safety, are the processes of reduction that occur in cathode materials, resulting in the production of oxygen and the partial hydrolysis of lithium hexafluorophosphate with the release of hydrogen fluoride [32]:

$$O_{2}+electrolyte\;\to \;{CO}_{2}+H_{2}O+∆H$$

$$Li\left({Ni}_{0.6}{Co}_{0.2}{Mn}_{0.2}\right)O_{2}+\;{CO}_{2}\to \;{Li}_{2}{CO}_{3}+Ni+Co+Mn+O_{2}$$

$$C+O_{2}\to {CO}_{2}+∆H$$

$$2Ni+O_{2}\to 2NiO$$

$$4NiO+O_{2}\to {2Ni}_{2}O_{3}$$

$$2Cu+O_{2}\to 2CuO$$

$$2Co+O_{2}\to 2CoO$$

$$2Mn+O_{2}\to 2MnO$$

$$4Li+O_{2}\to 2{Li}_{2}O$$

$$2Li+C_{3}H_{3}O_{3}\;(diethyl\;carbonate)\to \;{Li}_{2}{CO}_{3}+C_{2}H_{4}$$

$$2Li+C_{4}H_{6}O_{3}\;\left(dipropyl\;carbonate\right)\to \;{Li}_{2}{CO}_{3}+C_{3}H_{6}$$

$${LiPF}_{6}\to LiF+{PF}_{5}$$

$${PF}_{5}+H_{2}O\to 2HF+{POF}_{3}$$

It should be noted that the degradation mechanism of cathode and anode LIB materials varies depending on the specific cathode or anode material used. Each material undergoes distinct pathways that ultimately contribute to crystalline structure instability [33,34,35]. The state of charge (SOC) of the battery also plays a significant role in the combustion process, thermal runaway, and the possibility of explosion. Research results [36] indicate that the most intense thermal runaway occurs in a battery with a SOC of 75% rather than in a battery with a 100% SOC. This situation is caused by the significant mass loss in the battery with a 100% SOC. This leads to a significant amount of heat being dissipated into the cell, resulting in a lower maximum battery temperature [36]. Mao et al. [37] also demonstrated that as SOC increases, more metallic lithium is available in the anode. This element can react with the electrolyte, leading to the formation of more flammable gases. Furthermore, higher SOC levels lead to a higher heat of combustion of the gas mixture, which in turn increases the intensity of thermal runaway and combustion [37].

Due to the fact that the combustion reaction can be sustained by oxygen released during reactions occurring at the cell’s cathode, it is extremely difficult to completely extinguish a burning cell, which can burn without access to oxygen from the air, reaching temperatures exceeding 1000 °C [38]. This nature of the reactions complicates the selection of appropriate extinguishing agents. Other factors include cooling efficiency and emission levels, which can pose a risk to the environment and those involved in the incident. For example, it was found that the use of water mist can intensify the electrolyte hydrolysis reaction, leading to an increase in the peak concentration of hydrogen fluoride in the air [39].

Analysis of the extinguishing agents used showed that no smoke was observed when using quartz sand, and there were no traces of embers after the cylindrical lithium-ion battery was discovered. Minimal smoke emission and no traces of embers characterized the system in which exfoliated vermiculite was used for extinguishing. However, with extinguishing granules, intense smoke emission and numerous ember clusters were visible. Therefore, we analyzed the volume of dust emissions and the qualitative and quantitative characteristics of the hazardous substances emitted during the fire and its extinguishing. The analyses revealed the emission of large amounts of the inhalable fraction of total dust, metals, and PAHs (Table 3 and Table 4).

Analysis of the dust samples showed that the largest amounts of dust and PAHs were generated in a battery fire where exfoliated vermiculite was used to extinguish the fire. It should be noted that this material is produced by high-temperature firing (400–1000 °C) of raw vermiculite, a clay mineral from the aluminosilicate group, i.e., hydrated magnesium aluminosilicate. It has a loose, friable, and porous structure, and increases its volume several-fold under the influence of high temperatures. It is characterized by a stable layered structure and is resistant to aging and degradation. It is a natural and non-toxic material, chemically inert, and biologically sterile. Importantly, this substance is not flammable and does not support combustion. However, during a fire, depending on its composition and production process, toxic combustion products such as carbon oxides and other thermal decomposition products can be produced, which can pose a health hazard [40]. All these factors may lead to the formation of a continuous, insulating layer to be unable to form. This, in turn, can cause an additional combustion reaction, which would likely result in an increase in total PAH emissions from the battery fire involving vermiculite as the extinguishing agent, which were the highest, at 0.202 mg/m^3^. The highest levels of naphthalene, acenaphthylene, and fluorene were also detected in the air for this system. The lowest emissions of PAH compounds and total PAHs were observed for the battery-fire extinguishing granulate system. Similar correlations were observed for the analysis of the inhalable dust and metal emissions (Table 4).

The presence of metals in combustion products is a consequence of the substances used in battery construction, as well as fire extinguishing agents. Currently, intensive research is underway on the use of various lithium compounds, such as hypochlorite (VII) or organic salts, in battery construction [41]. The use of solid electrolytes based on metal oxides with complex structures and polymer electrolytes has become widespread. Graphite or metallic lithium is used as an anode. This cell structure suggests that the main components of fire fumes and gases, in addition to carbon dioxide, will be other products of incomplete oxidation of organic matter, such as carbon monoxide, formaldehyde, graphite-containing soot, hydrogen fluoride, and oxides of metals comprising the electrode and electrolyte, i.e., lithium, nickel, manganese, and cobalt. The results of analyses of dust generated during the battery fire indicate that the highest respirable dust fraction and the metals and zinc oxide contained in its structure in the air were recorded for the battery-exfoliated vermiculite system, while the lowest was recorded for the system in which fire extinguishing granules were used to extinguish the fire. The large amounts of aluminum observed in this system may be due to the release of this element from vermiculite, a material composed of aluminosilicates. Large amounts of cobalt, lithium, manganese, nickel, and lead were also observed for this system. Emissions for other metals were comparable. The quartz sand and fire extinguishing granules, which are characterized by a high silica content and a higher bulk density than vermiculite, reduced metal and PAH emissions into the air.

It should also be added that if the fire duration is prolonged, it will result in the emission of larger amounts of pollutants into the air, including heavy metals and alkali metals, semi-volatile organic compounds such as PAH, exceeding the permissible concentrations in the working environment (Table 5).

Because the amount of emitted substances depends on many factors (such as the size of the fire, type of battery combusted, ventilation efficiency, etc.), the obtained results can only be representative of small-scale fires in confined spaces, such as “prosumer” installations like small BESS in individual home basements or garages. In industrial-scale installations, the concentration of released chemicals will be significantly higher. This study is valuable due to the increasing popularity of installations of similar small systems in homes, and therefore allows a better understanding of the various risks associated with their use.

In an air sample collected during fire extinguishing with granulate, cobalt was observed at a level similar to the hygiene standard in Poland and the United States (0.02 mg/m^3^). In France and Germany, this value is considered to exceed applicable hygiene standards. Analyses revealed that permissible concentrations of cobalt and nickel in the air in the test room during and immediately after extinguishing with sorbent and granulate were exceeded. The test results are qualitatively consistent with those available in the literature [48,49,50], confirming the emissions of metals, including nickel, cobalt, aluminum, manganese, and lithium, into the environment. Due to the aforementioned reasons, quantitative comparison is not possible, as confirmed by the results of Claassen et al. [51]. These studies indicate that LCO (lithium cobalt oxide) battery fires were characterized by higher Li and Co emissions than LFP (lithium iron phosphate) battery tests. A database of air pollutant emissions based on battery type, size, and charge level, relative to the conditions in a given area or room volume, would allow for the development of tools necessary for proper and safe firefighting operations. However, first, it is necessary to establish the experimental conditions (including the construction of the test stand, distance between sensors, fuel type, height of the test room, ventilation, etc.), which would allow for a comparison of results and the development of appropriate solutions to minimize risks.

Due to these results, it is recommended that work in soot-contaminated rooms after battery extinguishing be performed using personal protective equipment (PPE) such as an FFP2 or FFP3 mask, after first checking the hydrogen fluoride concentration in the air using an electrochemical meter, which should be no more than 0.5 mg/m^3^. Short-term work (up to 15 min) is permitted at concentrations below 2.0 mg/m^3^. It should be noted that dust masks do not protect against chemical hazards in the form of gases, and the only effective protection against higher concentrations of hydrogen fluoride is a breathing apparatus with a full-face mask.

### 3.3. PAHs in Fire Debris

Fire residues, along with substances used as extinguishing agents, can be a source of environmental contamination. Therefore, it is essential to take measures to minimize the risk of environmental contamination. When using water or water-based extinguishing agents, it is important to consider the possibility of violent reactions between water and alkali metals, as well as the formation of fire effluent, which should be captured and neutralized. In the case of solid extinguishing agents, such as quartz sand, it is possible to minimize pollutant emissions by collecting residues and even recovering metals contained in fire residues. Metals such as lithium, cobalt, and cadmium are valuable raw materials that can be reused in the production of subsequent cells through the recycling process [52].

The results of analyses of fire residues in terms of PAH content showed that the smallest amounts of this group of pollutants were found in quartz sand, while in samples taken from systems where sorbent and extinguishing granules were used for extinguishing, the pollutants were approximately 10 and 100 times higher, respectively (Table 6).

PAH compounds and total PAHs indicate that the highest concentration of contaminants is found in the fire residue where extinguishing granules were used, while the lowest concentration is found in the quartz sand. This is likely due to the temperature of the fire site and the presence of embers (Table 1). PAH formation is favored by high temperatures and limited oxygen access, resulting in incomplete combustion, also known as smoldering fires [53]. The time and temperature required to melt the granules and create a layer insulating the cell result in the release of the highest concentration of contaminants into the extinguishing agent residue. However, this effect may be less significant in more advanced fires occurring at higher temperatures, where all available organic matter has already been burned off. In the case of quartz sand, in addition to the low temperature and absence of embers, the small amount of PAH bound in the residue may also be due to the dense packing and polar nature of the Si-O bonds, which provide a compact hydrophilic surface and prevent PAH deposition in the crystal lattice or on the grain surface. However, this process requires further work to determine the precise mechanism.

The results of the study conducted by Held et al. [54] also showed high levels of both PAHs and metals on surfaces up to 3 m from the fire site. Elevated levels of PAHs, approximately 250–300 μg/m^2^, were detected on collector plates and 120 μg/m^2^ on textiles, indicating that background levels for uncontaminated surfaces were exceeded by approximately 50 and 20 times, respectively. PAH compounds are toxic and carcinogenic substances; therefore, it is essential to monitor their levels at the fire site, where they often remain and migrate over long distances, causing environmental contamination.

It is also important to note that the fire extinguishing methods used to prevent thermal runaway and its spread in batteries, as well as in products in a given type of battery, require a specific approach. For example, in the case of electric and hybrid cars, the materials from which the vehicles are constructed, the components, and the type of fuel used must also be taken into account [20]. All these factors mean that the fire extinguishing process should be tailored to the device involved in the fire.

Currently, research and development are underway worldwide to replace lithium batteries with other batteries using cheaper materials for use in industrial energy storage systems. One promising alternative is the use of sodium cells. While they do not offer comparable energy densities to lithium-ion cells, they can be manufactured at a significantly lower cost without the use of less-readily available lithium [55]. Such cells can be used in stationary systems where mass and stored energy density are not key parameters of the energy storage system. The lower stored energy density also reduces the risk of ignition of these cells. If such a cell were to ignite, the pollutant profile released into the environment could differ from that of lithium-ion batteries.

## 4. Conclusions

Lithium-ion batteries have a wide range of applications in the economy and society. Their use has enabled the development of mobile telephones and electric cars. However, each product should be equipped with appropriate safety features, such as minimizing the likelihood of fire and providing access to extinguishing agents that enable heat capture and rapid cooling of the system, while minimizing the risk of environmental contamination and the threat to the health and lives of organisms. Therefore, the search for inexpensive, easy-to-use, and effective extinguishing agents is a crucial direction for the development of safety in various areas of the energy industry.

Extinguishing effectiveness tests indicate that the use of solid extinguishing agents such as quartz sand, natural sorbent (exfoliated vermiculite), and extinguishing granules varies in effectiveness in terms of cooling rate and produces varying levels of metal, dust, and PAH emissions. Extinguishing effectiveness tests showed that quartz sand was the best extinguishing agent tested. Extinguishing granules proved to be the least effective agent used in the test fires. The time and temperature required to melt the granulate and create an insulating layer on the cell resulted in the release of the largest amount of pollutants into the extinguishing agent residue. However, this effect may be less significant in the case of more advanced fires occurring at higher temperatures, where all available organic matter has burned out. The fine grains tightly cover the battery, allowing for rapid extinguishment and limiting the production of some toxic substances.

The natural sorbent tightly covered the burning battery, which, like with quartz sand, allowed for extinguishing the fire. However, during the process, a significant amount of toxic substances escaped from beneath the sorbent layer. The emission of pollutants in the form of metals and PAH compounds was similar. The system using extinguishing granulate as the extinguishing agent recorded the lowest pollutant emissions and the highest amount of PAH compounds accumulated in the fire residue. However, in the case of the system using vermiculite as the extinguishing agent, the highest PAH and metal emissions were recorded, while the amount of PAH in the fire residue was relatively low. Of the extinguishing agents used, considering cooling efficiency, the temperature of the fire site, the presence of embers, and the level of emissions of hazardous substances such as metals and PAHs, quartz sand was the most effective. However, further research is necessary, covering not only various batteries but also products in which batteries are used, including electric cars.

Research should address both the method of agent administration in relation to cooling efficiency, the level of pollutant emissions, and the contamination of the fire site, along with the possibility of metal recovery.

## Figures and Tables

**Table 1:** The Pearson correlation coefficients (R) for the standard curves and the relative standard deviation (RSD) for the individual analytes during the determination of PAHs.

PAH	Pearson Correlation Coefficient R	RSD [%]
Naphthalene	0.9999	2.15%
Acenaphthylene	0.9996	6.12%
Acenaphthene	0.9991	8.17%
Fluorene	0.9984	12.47%
Phenanthrene	0.9984	12.19%
Anthracene	0.9978	11.70%
Fluoroanthene	0.9977	11.86%
Pyrene	0.9968	12.67%
Benzo(a)anthracene	0.9972	13.27%
Chrysene	0.9969	10.54%
Benzo(k)fluoroanthene	0.9965	10.08%
Benzo(b)fluoroanthene	0.9987	10.08%
Benzo(a)pyrene	0.9948	8.09%
Indeno(1,2,3-cd)pyrene	0.9947	14.20%
Dibenzo(a,h)anthracene	0.9937	14.95%
Benzene(g,h,i)perylene	0.9925	14.83%

**Table 2:** Fire extinguishing effectiveness test results.

Extinguishing Agent	Fire Extinguished [YES/NO]	Fire Site Temperature [°C]	Observations
Quartz sand	YES	<100	After filling, the flame was immediately extinguished. Explosions were heard inside the tray. No smoke was observed. After the battery was discovered, no trace of embers was found (Figure 5a).
Exfoliated vermiculite	YES	<180	After completely covering the battery, the flame was immediately extinguished. Explosions were heard inside the tray. A minimal amount of smoke was noted. Upon uncovering the battery, no trace of embers was found (Figure 5b).
Fire extinguishing granules	YES	<500	After the battery was completely covered, the flame was immediately extinguished. Explosions were heard inside the tray. Heavy smoke was noted. Upon uncovering the battery, numerous clusters of embers were observed (Figure 5c).

**Table 3:** Results of analyses of PAH compounds in air samples collected during fire tests, depending on the extinguishing agent used (in [mg/m^3^]).

PAH	Inhalable Fraction		
	Quartz Sand	Exfoliated Vermiculite	Fire Extinguishing Granules
Naphthalene	0.0651	0.1353	0.0343
Acenaphthylene	0.0198	0.0296	0.0103
Acenaphthene	0.0004	0.0008	0.0004
Fluorene	0.0046	0.0096	0.0037
Phenanthrene	0.0096	0.0161	0.0057
Anthracene	0.0013	0.0027	0.0009
Fluoroantennae	0.0011	0.0035	0.0024
Pyrene	0.0006	0.0023	0.0019
Benzo(a)anthracene	<0.0001	0.0003	0.0005
Chrysene	<0.0001	0.0002	0.0005
Benzo(k)fluoroantennas	0.0001	0.0003	0.0003
Benzo(b)fluoroanthenes	0.0001	0.0003	0.0003
Benzo(a)pyrene	<0.0001	0.0002	0.0003
Indeno(1,2,3-cd)pyrene	<0.0001	0.0002	0.0002
Dibenzo(a,h)anthracene	<0.0001	0.0002	0.0001
Benzene(g,h,i)perylene	<0.0001	0.0001	<0.0001
TOTAL:	0.103	0.202	0.062

**Table 4:** Analysis of the inhalable fraction of total dust and metals contained in dust in air samples collected during fire tests, depending on the extinguishing agent used (in [mg/m^3^]).

Analyte	Inhalable Fraction Quantity		
	Quartz Sand	Exfoliated Vermiculite	Fire Extinguishing Granules
Soot dust	2.77	3.13	2.25
including:			
Zinc oxide	<0.021	<0.021	<0.021
Aluminum	<0.021	0.11	0.04
Cadmium	<0.0016	<0.0016	<0.0016
Cobalt	<0.01	0.06	0.02
Lithium	<0.01	0.12	0.03
Manganese	<0.021	0.04	<0.021
Copper	<0.021	<0.021	<0.021
Nickel	<0.021	0.56	0.18
Lead	<0.021	<0.21	<0.021
Iron	<0.021	0.03	<0.021

**Table 5:** Permissible concentration of selected harmful factors in the work environment (in [mg/m^3^]).

Toxic Substance	Australia [42]	Japan [43]	France [44]	Germany [45]	USA [46]	Poland [47]
Dust	10	10	10	10	10	10
Aluminum	Al_s_—10 Al_g_—5	-	Al_m_—10 Al_wf_—5	-	Al_if_—10	Al_if_—2.5 Al_rf_—1.2
Cobalt	Co_df_—0.05	0.05	0.001	0.005	0.02	0.02
Lithium	LiOH—0.025	LiOH—1.0	LiOH—0.02 (STEL)	Li_soh_—0.2	LiOH—0.025	LiOH—0.01
Manganese	Mn_df_—1	Mn_if_—0.1 Mn_rf_—0.02	Mn_if_—0.2 Mn_rf_—0.05	Mn_if_—0.15	0.2	Mn_if_—0.2 Mn_rf_—0.05
Nickel	1	1	1	0.03	0.5	0.25
PAH	Coal tar (dust and vapor—benzene-soluble fraction)—0.2	N/A	Coal tar (dust and vapor—benzene-soluble fraction)—0.2	B(a)P—0.00007 Mixture—0.0007	Naphthalene—100 Phenanthrene—0.00888 Anthracene—0.00079 Pyrene—0.009 Chrysene—0.00327 B(a)P—0.00249	as the product of the concentrations of 9 carcinogenic PAHs and their carcinogenicity coefficients—0.002

s—solid; g—gas; m—metal; wf—welding fumes; if—inhalable fraction; rf—resp. fraction; df—dusts and fumes; soh—salts, oxides and hydroxides; STEL—Short-Term Exposure Limit; N/A—not applicable.

**Table 6:** The amount of PAH in fire residues depending on the extinguishing agent used (in [mg/m^3^]).

PAH	The Amount of PAH in the Extinguishing Agent Residues		
	Quartz Sand	Exfoliated Vermiculite	Fire Extinguishing Granules
Naphthalene	0.078	0.833	10.50
Acenaphthylene	0.040	0.000	2.97
Acenaphthene	0.011	0.066	0.89
Fluorene	0.000	0.275	27.87
Phenanthrene	0.159	2.453	66.24
Anthracene	0.158	0.436	14.41
Fluoroantennae	0.071	0.000	10.82
Pyrene	0.101	2.202	8.32
Benzo(a)anthracene	0.000	0.790	1.94
Chrysene	0.007	1.602	3.92
Benzo(k)fluoroantennas	0.000	0.000	0.96
Benzo(b)fluoroanthenes	0.000	0.000	0.96
Benzo(a)pyrene	0.000	0.091	0.33
Indeno(1,2,3-cd)pyrene	0.002	0.063	0.17
Dibenzo(a,h)anthracene	0.002	0.049	0.13
Benzene(g,h,i)perylene	0.002	0.043	0.09
TOTAL:	0.631	8.902	150.52

## Data Availability

The original contributions presented in this study are included in the article. Further inquiries can be directed to the corresponding author.
